# Fellowships, Grants, & Awards

**Published:** 2006-08

## Reproductive Genetics and Epigenetics (R21)

The purpose of this Funding Opportunity Announcement (FOA) issued by the National Institute of Child Health and Human Development (NICHD), National Institutes of Health (NIH), is to continue to support new studies on the genes and genetic and epigenetic mechanisms influencing sex determination, fertility, reproductive health and reproductive aging, and other topics in Reproductive Genetics and Epigenetics. Studies submitted under this FOA are expected to identify and characterize the relevant genes, determine their function in normal human reproduction and reproductive development, identify functional partners or pathways and the nature of the interactions, and further our understanding of the consequences of mutations or dysregulation for human reproductive health. Studies of animal models are integral to this effort and are encouraged along with studies involving human subjects.

With the completion of the human genome project, the focus of genetic research must shift to functional genomics. NICHD encourages scientists interested in reproduction to lead the way in determining the genes and their mechanisms of action involved in the development of the gonads, reproductive ducts, and genitalia, the processes of gametogenesis, normal and premature reproductive aging, and reproductive disorders such as infertility, cryptorchidism, endometriosis, and polycystic ovarian syndrome (PCOS). Studies on the genetic epidemiology of reproductive disorders might begin with the collection of large numbers of affected patients and their relatives for linkage analysis, association studies or quantitative trait loci (QTL) analysis. Studies using innovative statistical or technical methods are highly encouraged. We also encourage research into epigenetic mechanisms critical to reproduction, especially areas such as the establishment and maintenance of methylation patterns or imprinted loci in the early embryo, the timing, mechanisms, and role of genomic methylation in gametogenesis, the effects of assisted reproductive therapy (ART) on imprinting and genomic methylation, and the reproductive determinants and consequences of X-chromosome inactivation.

Reproductive genetics is a broad research area, and the topics discussed and listed below are not meant to be exclusive areas of interest, but rather a sampling of the types of problems that this FOA intends to address.

### The genetics of sex determination

Sex determination is the translation of the chromosomal sex (XX or XY) into the gender-appropriate internal and external reproductive structures. The initial events of sex determination are, therefore, genetically determined. Errors in the process can range in severity from complete sex reversal to gonadal dysgenesis or minor genital abnormalities. Sex determination, as an early embryological event, can help us address basic questions of the regulation of gene expression, cell-fate determination, and hormone signaling.

Approximately one in 1,000 newborns has some abnormality of genital and/or gonadal development. In many cases, gonadal dysgenesis is part of a larger pathologic syndrome, such as Frasier syndrome, Deny-Drash syndrome, or campomelic dysplasia, to name a few. The known genes involved in sex determination often act as growth and/or differentiation factors, and there is mounting evidence that they may be important in tumorigenesis in the gonads as well as other tissues.

Despite the identification of the Y-chromosome gene SRY as the testis-determining factor almost 15 years ago, the mechanisms and pathways of normal sex differentiation are still not well understood. In particular, although some downstream effects of SRY are known, such as cellular proliferation, Sertoli cell differentiation, and testis-specific vascularization, the direct transcriptional targets of SRY remain unknown. The factors regulating SRY expression remain unknown as well. While genes such as SOX-9, WT-1, DAX-1, DMRT-1, GATA4, FOG2, and SF-1, among others, contribute to sex determination, the nature and timing of their interactions remain unclear, and there are clearly other unknown genes to be identified. A further level of complexity arises with gene dosage effects, such as XY sex reversal caused by duplication of Dax-1.

Sex determination can be divided into steps consisting of establishment of the bipotential gonad, formation of the primordial gonad, and differentiation of the gonad. Many of the sex determining genes act in multiple steps, but SRY mainly functions in shaping the primordial gonad into a testis. However, the classic view of SRY as a switch that confers maleness is an over-simplification as illustrated by the enormous potential for ambiguity in sex determination, and by evidence suggesting that steps in testis development that were once thought to be tightly coordinated, such as mesonephric cell migration and Leydig cell differentiation, or the formation of testis cords and the inhibition of male germ cell meiosis, can occur independently of each other. Additionally, ovarian development may not be the passive default process it was once thought to be. Estrogen may be necessary to maintain the ovarian phenotype, as mice unable to make estrogen (ArKO mice) or bind estrogen develop patches of Sertoli and Leydig cells within their ovaries postnatally.

Germ cells play a critical role in the formation of ovaries, although testes can form in their absence. The germ cells migrate into the gonad through the gut, through a process which has yet to be fully characterized. The presence of meiotic germ cells is critical for the formation and maintenance of ovarian follicles, while in contrast, in males the testis cords surround the germ cells and meiosis is inhibited. Germ cell migration and the progression into meiosis are not well understood.

There is clear evidence that the genes involved in sex determination have important roles beyond gonadal fate. Some, such as WT-1, are expressed in common embryonic precursors to different organ systems. Mutations in FOXL2, a gene deleted in polled intersex goats, cause the human syndrome BPES that often includes premature ovarian failure. The antimullerian hormone, known as Amh or MIS, causes regression of the female duct system in normal males, and in adult males, MIS has inhibitory effects on both Leydig cells and testosterone production. Such examples clearly demonstrate that the continued study of sex determination will not only benefit those born with gonadal dysgenesis or ambiguous genitalia, but will also advance our knowledge of the physiology of the adult reproductive system, and the development and regulation of other organ systems.

Specific topics of interest include, but are not limited to: 1) identification of the target genes and processes regulated by SRY; 2) clarification of the functional interactions between sex determining genes; 3) cloning of genes at loci associated with sex reversal, in humans and other species, and elucidation of their function; these studies may entail the collection of affected families or animal models and careful phenotypic description; 4) determination of how germ cell migration and meiosis affect sex determination and gonadal development; 5) study of the genes and processes regulating the retention or loss of the Wolffian and Mullerian ducts; 6) comparing and contrasting mammalian and nonmammalian sex determination systems to better understand the common pathways and genes; 7) creation of new cell or tissue culture systems, or animal models (especially transgenic or knock-out mice), to precisely characterize the functions of sex-determining genes.

### Genes regulating fertility, reproductive health, and reproductive aging

Infertility is a major public health problem in our country, affecting 10–15% of couples, or about 2.5 million couples in the United States. The annual cost of services to diagnose and combat infertility is now estimated at over one billion dollars. In recent years, great advances have been made in medical and surgical treatments for infertility caused by hormonal or structural defects. However, 30% of couples are infertile due to idiopathic or genetic causes, and they may suffer through failed conventional treatments before resorting to assisted reproductive technologies (ART) to conceive their biological children. Given the known and potential problems associated with the use of ART, it is essential that we focus our efforts on identifying and treating the underlying causes of infertility.

Studies of human infertility and studies using animal models have revealed many single gene mutations that cause infertility and new phenotypes continually appear in the literature. Each new gene teaches us more about the intricate pathways that contribute to normal fertility and may suggest leads for contraceptives. Epidemiological and family studies of human infertility are now feasible with the advent of genetic databases and new statistical techniques.

The most common identifiable cause of human male infertility is Klinefelter’s syndrome, occurring in 1 in 400 live births. The Klinefelter’s XXY genotype disrupts testis development and, in combination with high levels of meiotic nondisjunction, low sperm counts and infertility ensue. The Klinefelter’s phenotype, along with data showing exclusive expression of several X-chromosome genes in the testes, suggests that the X-chromosome figures prominently in testis physiology. Clearly, loci on the Y-chromosome are also critical to male fertility. Deletions within the male specific region of the Y-chromosome, previously referred to as the non-recombining region, are also a common genetic cause of spermatogenic failure in men. Mutation of specific genes within the AZF (azoospermia factor) regions of the Y-chromosome, most notably DAZ, severely disrupts spermatogenesis. The recent mapping of the male specific region of the Y-chromosome suggests that gene conversion (nonreciprocal recombination), while conserving important testis gene function on the Y-chromosome through evolution, may also predispose to deletions that abolish spermatogenesis.

Less dramatic mutations can also render males infertile. Disruption of the action of hypothalamic hormones can delay or prevent puberty, leading to oligospermia or azoospermia. Mutations causing both the X-linked and autosomal dominant forms of Kallmann’s syndrome (hypogonadotropic hypogonadism and anosmia), which is more common in males, were recently identified (KAL-1 and FGFR1, respectively). Similarly, mutation of the beta-subunit of the gonadotropin FSH also causes infertility by compromising spermatogenesis. Even when spermatogenesis proceeds smoothly, infertility can result if the chromatin is incorrectly packaged into the sperm head. Mutations that abolish the function of the transition proteins or the protamines that compact sperm chromatin cause infertility. The sperm mitochondrial genome also contributes to fertility. For example, absence of the common form of the POLG allele, encoding a mitochondrial DNA polymerase, is associated with infertility in men.

Genetic conditions in which the testes themselves are normal, but the male tract is affected, can render men infertile. Mutations in CFTR (the gene causing cystic fibrosis) can cause congenital bilateral absence of the vas deferens, seen in 1% of infertile men. Cryptorchidism is the most common defect of newborn boys, affecting 2–3%. Strong evidence demonstrates a genetic component to cryptorchidism. Mutation of the genes encoding either INSL3 (insulin-like hormone) or its receptor GREAT/LGR8, compromises the transabdominal phase of testicular descent, causing cryptorchidism which, if uncorrected, will result in infertility. However, the known mutations explain only a minority of cases of cryptorchidism, suggesting the involvement of other genes and pathways.

The identification of genetic causes of female infertility lags behind, possibly because the female reproductive system is more complex than the male system. Finely tuned cyclic fluctuations in hormones coordinate the follicular development, ovulation, and uterine receptivity for implantation, the components that comprise a normal menstrual cycle. This complexity suggests that there are hundreds of genes, each contributing a small effect on female fertility.

Genes involved in regulating the hypothalamic–pituitary–ovarian axis are obvious candidates for female infertility and, while mutations have been reported in the genes encoding FSH-beta and the LH receptor, and the genes associated with Kallmann’s syndrome have been identified, these mutations explain only a tiny proportion of cases of female infertility. However, work in highly prolific sheep has identified genes controlling ovulation rate and fertility, as well as ovarian development, which may lead to better understanding of infertility in women. In some breeds of ewes, naturally occurring mutations of genes encoding key players in the transforming growth factor beta signaling pathway increase ovulation rate and twinning. Conversely, homozygous mutation of the gene encoding the TGF signaling molecule BMP15 (GDF9B) causes sterility in the same breed of sheep. Such studies suggest new candidate molecules and pathways to study in human fertility.

The disruption of early embryonic development may be an underestimated cause of infertility. Mammalian oocytes store products necessary for the very early stages of development, until the embryonic genome is activated. Deletion of maternal oocyte products such as MATER, DNMT1o, and Npm2 arrests embryo development and leads to female infertility or subfertility in knockout mice. It is not known if mutations in these genes, or insufficient levels of their products, are a cause of human infertility.

Reproductive diseases such as endometriosis and polycystic ovarian syndrome are common and can be quite debilitating. Recent research indicates genetic components to these disorders; identification of causative or modifying genes would be of enormous benefit. Both diseases are likely to involve complex interactions between gene products and environment rather than single major genes. Polymorphisms in the insulin gene, the gene CYP11a, and the androgen receptor gene have been associated with hyperinsulinemia and hyperandrogenism in PCOS. Similarly, alterations in the estrogen receptor gene, genes encoding products involved in detoxification, homeobox genes, and the LH-beta gene, have been associated with a small number of cases of endometriosis. Comparative genomic hybridization and gene chip studies of endometriosis have revealed candidate regions and patterns of altered gene expression, but no major genes as yet.

Because of the sharp decline in female fertility with age and the increasing number of women who opt to have children later in life, the incidence of infertility is growing. Data from animal models and some human syndromes indicate that the timing of reproductive aging, in a continuum from premature ovarian failure to early menopause and normal menopause, may have genetic components. The genes and mechanisms contributing to reproductive aging have not been well characterized. Given the social trend to delay starting a family and the concerns about the prolonged use of hormone replacement therapy for menopause, understanding the mechanisms of reproductive aging is a high priority.

Premature ovarian failure (POF), defined as the cessation of menstruation before the age of 40, affects approximately 1% of women. Most cases of POF are assumed to be genetic and insight into this condition may help us better understand the variation in normal ovarian aging as well. Although the mechanism is not known, mutations in the gene encoding the FSH receptor are a rare cause of POF. Women carrying the fragile X premutation have a greater risk for premature ovarian failure, Mutation in a forkhead transcription factor, FOXL2 (3q23), causes autosomal dominant POF due to follicle depletion in some women affected with the syndrome BPES. FOXL2 mutation results in ovarian phenotypes ranging from streak ovaries to otherwise normal ovaries that lack adequate follicles. Mice lacking FOXO3A, a distant relative of FOXL2, show early depletion of ovarian follicles and sterility shortly after sexual maturity. Other causative genes for POF in women, and perhaps protective genes or alleles, remain to be identified.

The accumulation of meiotic errors in aging oocytes contributes strongly to the age-related decrease in women’s fertility and the increased risk for chromosomal abnormalities in children born to older mothers. This may be due to the unusual robustness of oocytes to proceed through meiosis despite flaws in the process; there are multiple examples of greater tolerance of meiotic defects in oogenesis as compared to spermatogenesis. For example, male germ cells are unable to progress through meiosis when the synaptonemal complex, which helps to hold homologous chromosomes together during meiosis, is compromised. While male mice bred to lack synaptonemal complex protein 3 are infertile, female SCP-3 knockout mice, though subfertile, are able to reproduce. Because the phenotype of subfertility due to embryo wastage becomes more severe with age, these mice may be a good model system not only for delineating the differences in meiosis in male and female gametes, but also for delineating the interactions between infertility and aging.

The phenomenon of reproductive aging in men, or decreased fertility with male age, is under debate and definitive studies are needed. Studies in old male rats demonstrate decreased fertility and an increased risk of siring abnormal offspring. Mutation rates appear to increase with age in male gametes and some genetic diseases, including both recessive X-linked and autosomal dominant conditions, demonstrate a paternal age effect, suggesting that the process of spermatogenesis does change with age in men. This is a phenomenon that needs further characterization and mechanistic study.

Specific topics of interest include, but are not limited to: 1) identifying specific Y-chromosome genes responsible for oligospermia or azoospermia, and establishing their functions in spermatogenesis; 2) identification of major genes, gene interactions or QTLS involved in regulating female fertility or ovarian or uterine function; 3) investigations of the heritability of infertility in offspring conceived through ART; 4) studies of the genetic mechanisms that establish the pool of primordial follicles and subsequent follicle development or loss; 5) identification of the gene mutations underlying inherited disorders of the reproductive organs or tract, such as PCOS, endometriosis, premature ovarian failure, and cryptorchidism, using candidate gene approaches as well as genetic epidemiology and linkage and/or association studies; 6) studies to elucidate the processes and mechanisms of the condensation and decondensation of the paternal and maternal genomes during gametogenesis and embryogenesis; 7) studies of the mechanisms responsible for the accumulation of meiotic errors in aging oocytes and identification of factors that impede or advance the process; 8) studies of similarities and differences in male and female meiosis, and how those contribute to the differential tolerance for meiotic errors; implications for fertility and contraception.

### Genomic imprinting and X-chromosome inactivation

The wealth of gene sequence data generated by the Human Genome Project will significantly improve our ability to detect and treat genetic diseases. However, diseases caused by epigenetic defects, such as improper gene methylation or improper X-chromosome inactivation, clearly demonstrate that in addition to a normal gene sequence, the timing, specificity, degree of gene expression, and even the parental origin of an allele are critical to normal human development and continued health. The epigenetic processes of imprinting and X-inactivation are intimately tied to reproduction, as the patterns are established during gametogenesis and embryogenesis, and they may in turn affect embryogenesis, gonadal/genital development, and fertility.

Imprinting is the phenomenon whereby one of the two autosomal alleles is preferentially expressed, dependent on its parental origin. Current estimates suggest that > 1% of all human genes are imprinted. Imprints are thought to be encoded by gene methylation patterns that differ between the maternally and paternally derived alleles. Parental imprints from the previous generation are erased in the germ cells at an early stage of development and new sex-specific imprints are established. This appears to occur before the onset of meiosis in male germ cells, but maternal imprints are established later, in growing oocytes arrested at the diplotene stage. Interestingly, the imprints are not all imposed together, as different genes are marked at various stages of oocyte growth. Although a genome-wide wave of demethylation occurs before implantation and de novo methylation reestablishes the pattern shortly after implantation, the core regions of the imprinted genes are somehow protected from these changes. Imprinting centers may play a role in the establishment and maintenance of the appropriate parental imprint, although the mechanism of such events remains unclear. Many imprinted loci encode anti-sense transcripts that have been implicated in the initiation of genomic imprinting, as well as X-chromosome inactivation.

Many key molecules regulating genomic methylation and transcriptional silencing have been identified. Methylation generally silences allele expression, as methyl-CpG-binding proteins such as MeCP2, bind to methylated DNA and recruit his-tone deacetylases. Hypoacetylated DNA is presumably inactive because it is conformationally inaccessible to the transcription machinery. The establishment and maintenance of DNA methylation are regulated by the DNA methyltransferases (Dnmt). Dnmt3A and Dnm3B function in *de novo* methylation, while Dnmt1 maintains methylation after each round of replication. Deficiency of Dnmt1 is lethal to embryos due to genome-wide demethylation. In contrast, the oocyte-specific form, Dnmt1o, seems to act only on certain genes and only at the eight-cell stage. Dnmt3L is required for the establishment of imprints during oogenesis, but is not necessary for the maintenance of paternal imprints during embryogenesis. BORIS, a paralog of CTCF, may participate in the erasure of parental methylation marks in the male germ line. More studies are needed to determine how the methylation and demethylation machinery correctly recognizes imprinted regions, discriminates between the maternal and paternal marks, and establishes or maintains the appropriate methylation patterns during gametogenesis and early embryogenesis.

Methylation of histones, in addition to DNA methylation, may regulate gene expression and the read-out of these types of methylation signals remains unclear. In mice lacking the polycomb group gene Eed, a subset of paternally repressed genes is improperly activated and expressed. Such data suggest that other transacting factors form an additional layer of regulation of the expression of imprinted genes.

Several human syndromes, such as Rett syndrome, ICF, Beckwith-Weidemann syndrome, Prader-Willi syndrome, and Angelman syndrome, are caused by defects in imprinting or in DNA methylation. Dysregulation of imprinted genes often manifests as abnormal growth of the fetus or placenta. One recently discovered example is the unknown locus on chromosome 19q13.4 that causes recurrent biparental complete hydatidiform molar pregnancies, as maternal alleles acquire paternal methylation patterns. Studies suggest that a failure of epigenetic reprogramming, as evaluated by methylation patterns, may underlie the extraordinarily high failure rate of cloning by nuclear transfer. The findings that cloned mouse embryos aberrantly express Dnmt1, while Dnmt1o fails to translocate to the nucleus, provide further support for this hypothesis. Culture conditions can also significantly and selectively alter the expression of imprinted genes, a finding that may be critical to human *in vitro* fertilization protocols. There is a trend among ART clinics to culture embryos for longer periods to enable selection of higher quality embryos; it is not clear if loss of imprinting occurs in such conditions and, if so, what effect it might have on the offspring. It seems likely that other more subtle phenotypes will be linked to defects in imprinting or DNA methylation/demethylation as well; exploration of these processes specifically in reproductive tissues is encouraged.

The inactivation of one X-chromosome in females is another type of gene silencing that acts as dosage compensation for the XX vs. XY genotype. Some critical X-linked genes "escape" inactivation and are expressed from both copies of the X-chromosome. Turner syndrome, resulting from a 45, X karyotype, clearly demonstrates the importance of genes on the second X-chromosome for fetal survival, as well as ovarian development.

There are two basic processes in X-inactivation: choice of which X-chromosome to inactivate, and implementation of the silencing. While recent studies show that X-inactivation has some mechanistic similarities to autosomal imprinting, X-chromosome inactivation in the embryo is usually random so that in each cell, the maternally and paternally-derived X-chromosome have an equal probability of inactivation. The molecule Xist, an X-encoded untranslated RNA, is the master regulator of X-chromosome inactivation. Xist is expressed only from the X-chromosome destined to become inactive (X-I). The Xist transcripts coat X-I in cis and soon after, histone 3 is methylated on lysine 9 on the inactive X. The X-chromosome that is destined to remain active (X-A) is protected from Xist by Tsix, the Xist antisense transcript. On X-A, histone 3 is methylated on lysine 4; this differential methylation suggests that a histone code may regulate the transcriptional status of the X-chromosome. The DNA of the inactive X-chromosome is hypermethylated and this is functionally significant as Dnmt1 mutant embryos fail to maintain random X-chromosome inactivation. Other events that mediate the silencing of the Xist-coated X-chromosome remain unknown. Recent data also suggest that there is active selection of both X-I and X-A, rather than one chromosome’s state being conferred by default.

Although the choice of which X-chromosome to inactivate is random in the embryo, it is imprinted in the extra-embryonic cells of mammals: the paternal X (Xp) chromosome is preferentially inactivated. The mechanisms for imprinted silencing of Xp in the extra-embryonic tissue and random X-chromosome inactivation in the embryo seem to be quite different. For example, Dnmt1 mutant embryos fail to maintain random X-chromosome inactivation in the embryo, but Xp is correctly inactivated in the extra-embryonic cells. Also, homozygous mutant eed mice initiate but fail to maintain imprinted Xp inactivation in the trophectoderm, but maintain normal random X-chromosome inactivation in the embryo itself, suggesting that eed functions only in maintenance of imprinted, but not random, X-chromosome inactivation.

Normal X-chromosome inactivation is essential to reproduction. Appropriate imprinted X-inactivation is critical to formation of the trophoblast and, ultimately, the placenta. Both heterozygous and homozygous Tsix knockout females are subfertile, with homozygous females showing a more drastic loss of fertility. Similar to imprinting defects in cloned embryos, cloned or *in vitro* embryos show disruption of dosage compensation of X-linked genes that may affect embryonic development.

The presence of skewed X-chromosome inactivation (XCI), usually defined as > 90% inactivation of a particular one of the pair of X-chromosomes, is increased in women with recurrent spontaneous abortion. In addition, women with skewed XCI and recurrent spontaneous abortion are more likely to have trisomic losses than women without XCI, but experiencing recurrent spontaneous abortion. Finally, deviations from random choice in X-chromosome inactivation can affect the relative expression of X-linked genes, many of which act in reproduction.

Transcriptional silencing of the X-chromosome (as well the Y-chromosome) occurs in males as well, just before meiotic prophase in spermatogenesis. The mechanism of male X-chromosome inactivation is likely completely different from that in the female because Xist mutation does not prevent the silencing in males. This remains a very poorly understood area.

Specific topics of interest include, but are not limited to: 1) identifying genes and mechanisms important in erasing and reestablishing genomic imprinting and genome-wide methylation during gametogenesis and early embryonic development; 2) characterizing the effects of manipulations of gametes or fertilized eggs, especially procedures commonly used in assisted reproductive technology, on gene methylation patterns, imprinting or X-inactivation; 3) investigation of defects in imprinting or methylation patterns in abnormal reproductive phenotypes including effects on game-togenesis, fertility, or gonadal differentiation and development; 4) description of the effects of mutations of the imprinting machinery in gametes and reproductive tissues, and on early embryonic development; 5) elucidation of the mechanism of the reversal of X-inactivation in XX primordial germ cells; 6) identification of the nature of the imprinting mark of the paternal X-chromosome and the mechanisms of imprinted X-inactivation in extra-embryonic cells; 7) studies of the biological significance and the mechanisms leading to X-chromosome inactivation in male meiotic germ cells; 8) studies of possible associations between skewed X-inactivation and various reproductive tract development and function, whether having protective or deleterious effects.

This funding opportunity will use the NIH Exploratory/Developmental Research Grant (R21) award mechanism. As an applicant, you will be solely responsible for planning, directing, and executing the proposed project.

This funding opportunity uses just-in-time concepts. It also uses the modular budget formats (see the Modular Applications and Awards section of the NIH Grants Policy Statement). Specifically, if you are submitting an application with direct costs in each year of $250,000 or less (excluding consortium Facilities and Administrative [F&A] costs), use the PHS398 Modular Budget component provided in the SF424 (R&R) Application Package and SF424 (R&R) Application Guide (see specifically Section 5.4, Modular Budget Component, of the Application Guide).

The R21 mechanism is intended to encourage new exploratory and developmental research projects and/or exploration of novel hypotheses and strategies. For example, such projects could assess the feasibility of a novel area of investigation or a new experimental system. These projects should be exploratory and novel, and distinct from the type of project supported through the traditional R01. For further information on the R21 mechanism, see: http://grants2.nih.gov/grants/funding/r21.htm.

Exploratory/developmental grant support is for new projects only; competing renewal (formerly competing continuation) applications will not be accepted. Up to two resubmissions (formerly revisions/amendments) of a previously reviewed exploratory/developmental grant application may be submitted. See NOT-OD-03-041, May 7, 2003.

Applicants must download the SF424 (R&R) application forms and SF424 (R&R) Application Guide for this FOA through Grants.gov/Apply.

Note: Only the forms package directly attached to a specific FOA can be used. You will not be able to use any other SF424 (R&R) forms (e.g., sample forms, forms from another FOA), although some of the Attachment files may be useable for more than one FOA. For further assistance contact GrantsInfo, 301-435-0714 (telecommunications for the hearing impaired: TTY 301-451-0088) or by e-mail: GrantsInfo@nih.gov.

Prepare all applications using the SF424 (R&R) application forms and in accordance with the SF424 (R&R) Application Guide (MS Word or PDF).

The SF424 (R&R) Application Guide is critical to submitting a complete and accurate application to NIH. There are fields within the SF424 (R&R) application components that, although not marked as mandatory, are required by NIH (e.g., the Credential log-in field of the Research & Related Senior/Key Person Profile component must contain the PD/PI’s assigned eRA Commons User ID). Agency-specific instructions for such fields are clearly identified in the Application Guide. For additional information, see Tips and Tools for Navigating Electronic Submission on the front page of Electronic Submission of Grant Applications.

The SF424 (R&R) application is comprised of data arranged in separate components. Some components are required, others are optional. The forms package associated with this FOA in Grants.gov/APPLY will include all applicable components, required and optional. A completed application in response to this FOA will include the following components: 1) SF24 (R&R) (cover component); 2) research and related project/performance site locations; 3) research and related other project information; 4) research and related senior/key person; 5) PHS398 cover page supplement; 6) PHS398 research plan; 7) PHS398 checklist; 8) PHS398 modular budget.

The application submission dates for this PA are available at http://grants.nih.gov/grants/funding/submissionschedule.htm. The complete version of this PA is available at http://grants.nih.gov/grants/guide/pa-files/PA-06346.

Contact: Susan Taymans, Reproductive Sciences Branch, Center for Population Research, National Institute of Child Health and Human Development, 6100 Executive Boulevard, Room 8B01, Bethesda, MD 20892-7510, 301-496-6517, fax: 301-496-0962, e-mail: Taymanss@mail.nih.gov.

Reference PA-06-346.

## Integrating Lung Genetics and Genomics in Human Populations (R01)

Over the past few years, compelling evidence (in mouse, yeast, and a few human studies) has demonstrated the power of integrating genotypic and gene expression data to accelerate gene discovery. Potentially, it represents a more powerful way to understand the relationship of genes to human complex traits. This will require linking expression array technology, genotyping technology, bioinformatics, and genetic statistics in innovative ways and applying these methodologies to clinical phenotypes to identify genes and predict clinical outcomes. Examples of this would be the use of expression profiles as the basis for phenotypes for linkage analysis or the use of expression profiles with SNP association data to predict clinical outcomes. Presently, these recent developments that accelerate gene finding have not been applied to the lung because investigators are exploring these as separate disciplines. Investigators know how to do one or the other of these types of experiments, but not both, hindering the integration of these approaches. This program announcement seeks to provide an important stimulus to bring these complementary fields together to more rapidly identify genes associated with lung disease and more efficiently identify the gene networks associated with health and disease in the lung.

This is important because it remains difficult to identify genes related to complex trait phenotypes in the lung. Utilizing expression and association together will make identifying genes and gene pathways much easier. It will be easier because expression is a complementary technology to association that can validate association findings and demonstrate novel relationships among genes. Since genes work together with other genes in pathways, finding the critical interrelationships is important. In addition, expression can be used to define subpheno-types of disease that can then be used for clinical prediction in association studies. Identifying these genes is the first step to developing models that will better predict disease natural history, disease risk, response to therapy, and risk of hospitalization. Prediction has proceeded faster in other disease areas than in lung disease, such as cancer, because of the use of gene arrays on tumor tissue. No lung disease area has yet combined arrays with genotyping and genetic association. If successful, it will fuel translational research that will bring the human genome project to the clinical arena much faster. Bringing expression and genetic association together in studies of lung disease will internally validate gene targets, enhance linkage and association analysis, assist in describing epistatic interactions, and define new pathways. Thus, it will advance the agenda for predictive medicine, which is one of the translational goals of the human genome project and an important goal of the NHLBI.

The completion of the human genome project and the development of high-throughput technologies such as gene-chip and rapid SNP genotyping greatly facilitate the search for genes that contribute to disease variability and disease risk. The purpose of this initiative is to stimulate innovative approaches to merge genetic and genomic techniques to find genes associated with human complex traits for lung disease.

Research topics to be addressed as part of this initiative include, but are not limited to: 1) studies that integrate linkage and association data with gene expression data to identify novel loci related to disease pathogenesis, disease severity, or response to drug therapy (pharmacogenetics); 2) studies that integrate gene expression data with cross-sectional and longitudinal quantitative or discrete clinical phenotypes as a means to identify novel gene targets and pathways, and to refine phenotype definitions based on expression profiles; 3) studies that develop clinical prediction models based on combinations of genetic (genotype) data with gene expression profiling; 4) genome-wide association results correlated with genome-wide expression results to determine overlap at the genome level; 5) studies that use genomic applications to confirm or validate the possible genotype-phenotype associations in complex traits; 6) studies that develop new statistical or bioinformatics approaches to the analysis of large amounts of genetic and genomic data.

It would be expected that investigators would have the population in hand, and either DNA or RNA or relevant cells to respond to this PA. However, collection of either DNA or RNA or cells would be permissible under this PA. Groups expert in genetics or genomics are encouraged to collaborate with each other to accomplish the goals of this PA. Since expression is tissue specific, investigators must demonstrate the relevance of the cells being used for expression to the lung disease of interest. These approaches are not exclusive and investigators are encouraged to consider novel methods as to how they would apply genetic (genotype) and genomic (expression) technologies to address important unsolved clinical questions of relevance to human lung disease.

This funding opportunity will use the R01 award mechanism. As an applicant, you will be solely responsible for planning, directing, and executing the proposed project.

This funding opportunity uses just-in-time concepts. It also uses the modular as well as the nonmodular budget formats (see http://grants.nih.gov/grants/funding/modular/modular.htm). Specifically, if you are submitting an application with direct costs in each year of $250,000 or less, use the modular budget format described in the PHS 398 application instructions. Otherwise follow the instructions for nonmodular research grant applications.

The PHS 398 application instructions are available at http://grants.nih.gov/grants/funding/phs398/phs398.html in an interactive format. Applicants must use the currently approved version of the PHS 398. For further assistance contact GrantsInfo, 301-435-0714 (telecommunications for the hearing impaired: TTY 301-451-0088) or by e-mail: GrantsInfo@nih.gov.

Applications must be prepared using the most current PHS 398 research grant application instructions and forms. Applications must have a D&B Data Universal Numbering System (DUNS) number as the universal identifier when applying for federal grants or cooperative agreements. The D&B number can be obtained by calling 866-705-5711 or through the web site at http://www.dnb.com/us/. The D&B number should be entered on line 11 of the face page of the PHS 398 form.

The application submission dates for this PA are available at http://grants.nih.gov/grants/funding/submissionschedule.htm. The complete version of this PA is available at http://grants.nih.gov/grants/guide/pa-files/PA-06-370.

Contacts: Susan Banks-Schlegel, Division of Lung Diseases, National Heart, Lung, and Blood Institute, Two Rockledge Center, Suite 10018, 6701 Rockledge Drive, Bethesda, MD 20892-7952 USA, 301-435-0202, fax: 301-480-3557, e-mail: Schleges@nih.gov; Dorothy Gail, Division of Lung Diseases, National Heart, Lung, and Blood Institute, Two Rockledge Center, Suite 10018, 6701 Rockledge Drive, Bethesda, MD 20892-7952 USA, 301-435-0222, fax: 301-480-3557, e-mail: Gaild@nhlbi.nih.gov.

Reference PA-06-370.

